# The effect of bile salt diet supplementation on genes related to fat metabolism in yellow-feathered broilers

**DOI:** 10.14202/vetworld.2022.911-918

**Published:** 2022-04-13

**Authors:** Zhenming Zhang, Baoan Ding, Hailian He, Jingge Wang, Xiongjie Liu, Jiahui Guo, Pengxiang Li, Stephen R. Madigosky

**Affiliations:** 1 Department of Animal Science, College of Agriculture and Animal Husbandry, Qinghai University, Xining, 810016, China; 2 Department of Environmental Science and Biology, One University Place, Widener University, Chester, Pennsylvania 19013, USA

**Keywords:** acetyl-CoA carboxylase, fat metabolism, fatty acid synthase, fatty acid transporter 4, sterol regulatory element-binding transcription factor 1, supplemental dietary bile salts

## Abstract

**Background and Aim:**

As a new feed additive, bile acid (BA) can promote the absorption and transport of lipids and fat-soluble vitamins. In recent years, BAs have been widely used in animal feed to promote fat absorption. Therefore, this study aimed to investigate the effect of bile salt supplementation in the diet of yellow-feathered broilers on messenger RNA (mRNA) expression of sterol regulatory element-binding protein 1 (SREBF1), fatty acid synthase (FAS), acetyl-coenzyme A carboxylase (ACC), and fatty acid transport protein 4 (FATP4).

**Materials and Methods:**

Four hundred and twenty commercial male chicks were randomly divided into seven groups (with four replicates per group and 15 chickens per replicate). They were fed diets supplemented with bile salts at 0, 1.5, 2.5, 3.5, 4.5, 5.5 mg/kg, and 2 mg/kg tylosin for 30 days. Changes in SREBF1, fatty acid transporter 4, FAS, and acetyl-CoA carboxylase genes in intestinal mucosa and liver of yellow-feathered broilers were determined using a quantitative fluorescence polymerase chain reaction.

**Results:**

mRNA expression of SREBF1, FAS, ACC, and FATP4 in the small intestine decreased in chicks fed diets supplemented with 3.5 and 4.5 mg/kg bile salts (p<0.05) compared with the control group on 7 days and 14 d. The mRNA expressions of SREBF1, FAS, ACC, and FATP4 in liver tissue decreased in chicks fed diets supplemented with 4.5 and 5.5 mg/kg bile salts (p<0.05) compared to the control group on 7 days. The mRNA expression of SREBF1, FAS, ACC, and FATP4 in the liver at 14 days and the small intestine on 21 days also decreased in chicks fed diets supplemented with 4.5 mg/kg bile salts (p<0.05) compared to the control group. When contrasted with the control group on day 21, the mRNA expression of SRWBF1, FAS, ACC, and FATP4 detected in the liver was lower in chicks fed diets supplemented with bile salts (p<0.05).

**Conclusion:**

The dietary supplementation of bile salts at 4.5 mg/kg effectively regulates the expression of fat metabolism genes, such as SREBF1, FAS, ACC, and FATP4 mRNA. At this concentration, bile salts promote fat catabolism, inhibit fat synthesis, and play an essential role in improving the fat deposition of broilers.

## Introduction

Bile acids (BAs) are synthesized from a unique pathway using cholesterol in the liver. They are specific and quantitatively important organic components in bile and play an important role in intestinal fat digestion and the absorption of fat-soluble vitamins [1]. Besides emulsifiers that promote lipid absorption, BAs have also been found to play an important regulatory role in various metabolic processes of the body as a signal molecule and an indispensable regulator in glucose and lipid metabolism [2].

In recent years, BAs have been widely used as innovative additives in animal feed to promote the absorption of fat, which serves as a primary mechanism in the catabolism of cholesterol in mammals. Evidence has suggested that restricted bile secretion in chicks, especially in the 1^st^ week after the hatching, reduces fat digestibility [3]. Parsaie *et al*. [4] have shown that adding bile salts to broiler diets can significantly increase weight. Broilers cannot secrete enough bile salts over time to satisfy fat digestion compared to adult chickens. Therefore, the digestion and metabolism of fat are improved in young chickens with dietary bile salts.

Bile salts and their derivatives have been recently used as a dietary supplement to promote efficient growth in chickens by improving fat digestion and absorption [5]. Lai *et al*. [6] show that the addition of BAs to the diet can effectively improve the growth performance of broilers and the quality of chickens by increasing the activity of lipoproteins and lipases in the duodenum. However, few studies explored the lipid metabolism of BAs in poultry.

Research shows that regulatory genes that control sterol regulatory element-binding protein 1 (SREBF1), fatty acid transport protein 4 (FATP4), fatty acid synthase (FAS), and acetyl-coenzyme A carboxylase (ACC) are all involved in fat metabolism [7]. Sterol element-binding proteins (SREBPs) can influence lipid homeostasis by regulating their target genes, essential for cholesterol and fatty acid metabolism [8,9]. The mature forms of SREBPs have transcriptional activity and are transferred to the nucleus, where they bind to the promoters of SREBPs target genes and take part in lipid metabolism [10]. Fatty acid transporter 4 (FATP4) is a member of the multigene family of fatty acid transporters and is considered the main fatty acid transporter for intestinal lipid absorption [11]. FAS is a multipurpose enzyme that catalyzes the synthesis of various fatty acids. These are largely formed in the liver, and nutritional signals control some lipid tissues and FAS expression [12]. Acetyl-CoA carboxylase (ACC) is an essential rate-limiting enzyme in fatty acid metabolism [13].

Therefore, this study aimed to investigate the effect of bile salt supplementation in the diet of yellow feather broilers to determine their role in regulating fat metabolism-related genes.

## Materials and Methods

### Ethical approval

All procedures outlined herein were in compliance with the national laws and regulations for animal experimentation and were performed in accordance with the oversight principles mandated by the Qinghai University Animal Care Committee for the care and use of experimental animals (Approval no. IACUC, SL-2020009).

### Study period and location

The study was conducted during June and July 2020. The study was conducted at the chicken farming facility at the Changlui Animal Husbandry Company in Huangzhong County on the Qing-Zang plateau in northwestern China, at an altitude between 2500 and 3000 m above sea level.

### Bird management and treatment

Four hundred twenty, 1-day-old yellow-feathered broilers vaccinated from Marek’s disease were randomly selected and divided into seven groups with four replicates per group (15 chickens per replicate) using a single factor experimental design. Precise amounts of bile salts (2 mg/kg tylosin and 0, 1.5, 2.5, 3.5, 4.5, and 5.5 mg/kg) were added to the diet of the experimental Groups C, A, B1, B2, B3, B4, and B5. C was the positive control group (tylosin), and A was the negative control group. Tylosin is a special antibiotic for livestock and poultry. It has wide antibacterial spectrum, fast absorption, good diffusion ability and growth promotion. As a feed additive, the rational use of tylosin can not only prevent livestock and poultry diseases and ensure the health of livestock and poultry, but also significantly promote the growth and development of livestock and poultry, especially for young and growing livestock and poultry. It can improve feed utilization rate, shorten feeding cycle and increase breeding economic benefits. Therefore, we selected tylosin as the positive control group. The BA doses studied by Parsaie *et al*. [4] and Lai *et al*. [6] were used to formulate BA doses for this study.

Diets (mash) were formulated to meet the National Research Council nutrient requirements, USA [14]. Chicks were fed experimental diets (Table-1) until 30 days of age (Table-1). The ingredients and chemical compositions of the diets were analyzed using AOAC procedures [15]. The birds were housed in wire-floored, stainless-steel cages, and kept indoors between 30°C and 32°C from 1 to 14 days, and at 24°C and 26°C from 15 to 30 days. Incandescent 5 lux bulbs provided a continuous source of light throughout the experiment. Feed and water were provided *ad libitum*.

**Table 1:** Composition of dietary ingredients fed to broiler chickens (The experimental diet based on NRC).

Ingredients (%)	A	C	Bile acid salt				

			B1	B2	B3	B4	B5
Corn	56.81	56.81	56.81	56.81	56.81	56.81	56.81
Soybean meal	28	28	28	28	28	28	28
Soybean oil	2	2	2	2	2	2	2
Wheat bran	5.3	5.28	5.285	5.275	5.265	5.255	5.245
Yeast powder	3	3	3	3	3	3	3
Limestone	1.96	1.96	1.96	1.96	1.96	1.96	1.96
Dicalcium phosphate	1.1	1.1	1.1	1.1	1.1	1.1	1.1
Salt	0.3	0.3	0.3	0.3	0.3	0.3	0.3
Methionine	0.23	0.23	0.23	0.23	0.23	0.23	0.23
Lysine	0.35	0.35	0.35	0.35	0.35	0.35	0.35
Threonine	0.08	0.08	0.08	0.08	0.08	0.08	0.08
Sodium sulfate	0.1	0.1	0.1	0.1	0.1	0.1	0.1
Microelement	0.12	0.12	0.12	0.12	0.12	0.12	0.12
Choline chloride (50%)	0.1	0.1	0.1	0.1	0.1	0.1	0.1
Baking soda	0.05	0.05	0.05	0.05	0.05	0.05	0.05
Premi×^1)^	0.5	0.5	0.5	0.5	0.5	0.5	0.5
Bile acid salt	0	0	0.015	0.025	0.035	0.045	0.055
Tylosin	0	0.02	0	0	0	0	0
Calculated							
Dry matter	84.36	84.34	84.35	84.34	84.33	84.32	84.31
ME (MJ/kg)	11.84	11.84	11.84	11.84	11.84	11.84	11.84
CP	19.55	19.5	19.55	19.55	19.55	19.55	19.54
EE	4.47	4.47	4.47	4.47	4.47	4.47	4.47
CF	3.81	3.81	3.81	3.81	3.81	3.81	3.80
Ash	2.80	2.80	2.80	2.80	2.80	2.80	2.80
Ca	1.09	1.09	1.09	1.09	1.09	1.09	1.09
P	0.64	0.64	0.64	0.64	0.64	0.64	0.64
Methionine	0.55	0.55	0.55	0.55	0.55	0.55	0.55
Lysine	1.45	1.45	1.45	1.45	1.45	1.45	1.45
Threonine	0.86	0.86	0.86	0.86	0.86	0.86	0.86

ME=Metabolizable energy, CP=Crude protein, EE=Crude fat, CF=Crude fiber, Ash=Ash content, Ca=Calcium, P=Phosphorus.

1) Fifty percent premixes contain complex enzymes at a concentration of 250 mg/kg phytase, 250 mg/kg Probiotics, 500 mg/kg Naxiseptide, 250 mg/kg Polysaccharide, 500 mg/kg Vitamin mixture 230 mg/kg, Microelement contain 8 mg/kg copper, 60 mg/kg iron, 60 mg/kg zinc, 60 mg/kg manganese, 0.15 mg/kg selenium, and 0.35 mg/kg iodine

### Sample collection

Chicks were randomly selected and sacrificed on days 7, 14, and 21 after 6 h of fasting (one bird per replicate) by cervical dislocation. Segments of approximately 5 cm of the duodenum (midpoint of the pancreatic loop), jejunum (midpoint of the jejunum), and ileum (after Meckel’s diverticulum) were obtained before the removal of the entire intestinal tract and cut longitudinally downward. All samples were placed in 2.0 mL Eppendorf tubes and frozen in liquid nitrogen. They were then placed in a −80°C freezer for long-term storage.

### Real-time quantitative polymerase chain reaction (PCR) analysis of gene expression

PCR methods were used to analyze gene expression through quantitative characterization of tissue samples. Total RNA was isolated from the duodenum, jejunum, ileum, and liver with Trizol reagent (Tiangen, Bejing, China). After determining RNA purity and concentration and verifying RNA integrity, the messenger RNA (mRNA) was reverse transcribed into complementary DNA (cDNA) using a commercial kit (PrimeScript RT Reagent kit, TaKaRa, Dalian, P. R. China). Genetic characterization of tissue samples was ascertained using a BIO-RAD CFX96 Touch Real-time PCR detection system (Bio-Rad, USA). The reaction mixture comprised 2 μL of cDNA, 0.6 μL of the forward and reverse primers, 10 μL of SYBR1 Premix Ex Taq (2×), and 6.8 μL of ddH_2_O. The reaction conditions were 95°C for 2 min, 40 cycles of 95°C for 15 s, and 58°C for 30 s. The relative mRNA expression levels of genes were calculated by the 2−DDCt method and normalized to the value of β-actin. The primer sequences used are listed in Table-2. Each sample was assayed in triplicate. The primer sequences were obtained according to previous studies [16,17], synthesized by Sangong Bioengineering Ltd. (Shanghai, China).

**Table 2:** Primer sequences used in the real-time polymerase chain reaction.

Gene^1^	Primer sequence	Orientation	Length
SREBF1	GCAGAAGAG CAAGTCCCTCAA	Forward	130
	GGAGCCTAC ATCCGAGGG	Reverse	
FATP4	ATACCTCTGG CACTACGGGAAT	Forward	117
	CATACATCAC ATCATCGGGTCT	Reverse	
FAS	AGAGGCTTT GAAGCTCGGAC	Forward	127
	GGTGCCTGA ATACTTGGGCT	Reverse	
ACC	TTGTGGCAC AGAAGAGGGAA	Forward	161
	GTTGGCACA TGGAATGGCAG	Reverse	
B-actin	AACACCCAC ACCCCTGTGAT	Forward	116
	TGAGTCAAGC GCCAAAAGAA	Reverse	

Gene 1 represents; 1SREBF1=Sterol regulatory element binding transcription, factor 1, FATP4=Fatty acid transporter 4, FAS=Fatty acid synthase, ACC=Acetyl-CoA carboxylase

### Statistical analysis

The relative expression levels of the target genes were calculated using the Livak method. Then, multiple comparisons were performed using Duncan’s Statistical Package for the Social Sciences 22.0 (IBM Corp., NY, USA). General Linear Model and the results were expressed as the mean ± standard deviation.

## Results

### Development changes of SREBF1 mRNA in yellow-feathered broilers

Development changes of SREBF1 mRNA in yellow-feathered broilers on days 7, 14, and 21 after adding dietary bile salts are shown in Table-3. Compared with the negative control group, the expression of SREBF1 mRNA in the duodenum decreased in B4 on 7 days and was lowered in B1, B2, and B3 at 14 days. Compared with the positive control group, B3 reduced the expression of SREBF1 mRNA at 14 days (p<0.05). The expression levels of SREBF1 mRNA in jejunum were reduced in B4 on days 7 and 14. A reduction occurred in B2, B3, and B4, while on day 21, a reduction was noted in B1, B4, and B5 (p<0.05) compared to the positive control. When these were compared to the positive control on 21 days, the expression of SREBF1 mRNA in the jejunum increased except for B1, B4, and B5. When the expression of SREBF1 mRNA was compared between the negative control and experimental groups in the ileum, a decrease was found in B4 on day 14, and B4, and B5 on day 21 (p<0.05). Comparison among the positive control with the experimental groups adding bile salts showed that the expression of SREBF1 mRNA decreased everywhere except B4. We noted an increase on days 14, and 21, B5 decreased the expression of SREBF1 mRNA (p<0.05). When the expression of SREBF1 mRNA was compared in liver tissue with the experimental groups, we noted a decrease in B4 and B5 on 7 days, B4 at 14 days, and the bile salt supplement group on 21 days (p<0.05). Comparisons between the positive control and bile salt groups showed increased activity on day 14 except B1, B2, and B4 (p<0.05). On day 21, B3 reduced the expression of SREBF1 mRNA (p<0.05).

**Table 3:** Relative expression of SREBF1 mRNA on 7, 14, and 28-day-old yellow feather broiler chickens.

Treatments	A	C	B1	B2	B3	B4	B5
7 days							
Duodenum	1.01±0.20^bc^	0.63±0.10^d^	1.81±0.33^a^	1.05±0.47^bc^	0.96±0.13^bc^	0.70±0.09^d^	1.10±0.09^b^
Jejunum	1.00±0.14^bc^	0.72±0.10^d^	1.71±0.14^a^	0.98±0.15^bc^	1.14±0.20^b^	0.82±0.20^d^	1.60±0.14^a^
Ileum	1.00±0.10^c^	0.81±0.15^c^	1.81±0.27^b^	3.74±0.48^a^	1.09±0.10^c^	0.93±0.26^c^	1.04±0.56^c^
Liver	1.01±0.06^c^	0.50±0.05^e^	1.74±0.10^a^	0.83±0.16^cd^	1.31±0.18^b^	0.79±0.10^d^	0.48±0.17^e^
14 days							
Duodenum	1.00±0.07^a^	0.47±0.06^c^	0.76±0.16^b^	0.70±0.12^b^	0.24±0.02^d^	0.88±0.12^ab^	0.82±0.18^ab^
Jejunum	1.00±0.25^b^	0.73±0.34^c^	2.14±0.36^a^	0.74±0.21^c^	0.66±0.12^c^	0.74±0.22^c^	1.28±0.16^b^
Ileum	1.00±0.08^bc^	0.67±0.06^cd^	1.62±0.27^a^	1.17±0.39^b^	0.82±0.05^cd^	0.58±0.21^d^	0.99±0.10^bc^
Liver	1.00±0.07^bc^	2.44±0.69^a^	0.79±0.07^bc^	1.00±0.05^bc^	1.69±0.05^ab^	0.46±0.08^d^	2.63±1.48^a^
21 days							
Duodenum	1.00±0.14^c^	0.97±0.50^c^	1.09±0.04^c^	0.76±0.15^c^	0.74±0.06^c^	2.13±0.20^b^	6.17±0.90^a^
Jejunum	1.00±0.17^c^	1.25±0.33^bc^	0.70±0.08^d^	2.77±0.19^a^	1.39±0.45^b^	0.66±0.06^d^	0.72±0.18^d^
Ileum	1.00±0.05^c^	0.73±0.11^d^	1.23±0.10^b^	1.49±0.22^a^	0.99±0.10^c^	0.83±0.05^d^	0.10±0.02^e^
Liver	1.00±0.03^a^	0.44±0.10^c^	0.45±0.26^c^	0.48±0.10^bc^	0.12±0.03^d^	0.29±0.17^cd^	0.70±0.22^b^

Means within columns with different superscript letters are different per P<0.05

### Development changes of FATP4 mRNA in yellow-feathered broilers

Table-4 shows the relative expression levels of FATP4 mRNA in yellow feathered broilers fed with dietary bile salt supplements on 7, 14, and 21 days of age. Compared with the negative control group, the expression of FATP4 mRNA in the duodenum decreased in B3 on day 14, and it decreased in B3, B4, and B5 on 21 days (p<0.05). Compared with the positive control group, all BA groups reduced the expression of FATP4 mRNA on 21 days (p<0.05). The levels of expression of FATP4 mRNA in jejunum were reduced in B1 and B3 on day 14 (p<0.05) compared to the control. When these were compared to the positive control on 21 days, the expression of FATP4 mRNA in the jejunum was reduced in B1, B3, B4, and B5 (p<0.05). When the expression of FATP4 mRNA was compared between the negative control and experimental groups in the ileum, a decrease was found in B4 and B5 on 7 days, B4 on day 14 and except for B2, B3, B4, and B5 on day 21 (p<0.05). Comparisons among the positive control with the experimental groups adding bile salts showed that the expression of SREBF1 mRNA decreased B4 on day 7. On day 14, decreased most groups except B1, B3, and B4, and on day 21, there was a decrease in the BA supplement group (p<0.05). When the expression of FATP4 mRNA was compared in liver tissue with the experimental groups, we noted a decrease in B4 and B5 on 7 days, B3, and B4 on 14 days and B2, B3 and B4 on 21 days (p<0.05). Comparisons between the positive control and bile salt groups showed B4 and B5 reduced on day 7. On day 21, except for B1, the other bile salt groups all reduced the expression of FATP4 mRNA (p<0.05).

**Table 4:** Relative expression of FATP4 mRNA on 7, 14, and 28-day-old yellow feather broiler chickens.

Treatments	A	C	B1	B2	B3	B4	B5
7 days							
Duodenum	1.00±0.30^c^	1.02±0.29^c^	1.50±0.20^b^	1.14±0.23^c^	1.82±0.07^b^	0.90±0.09^c^	3.62±0.32^a^
Jejunum	1.01±0.13^b^	1.22±0.20^b^	2.72±0.38^a^	1.29±0.35^b^	0.86±0.11^b^	0.99±0.29^b^	2.76±0.50^a^
Ileum	1.00±0.03^c^	0.87±0.12^cd^	1.00±0.10^c^	1.88±0.22^a^	1.40±0.26^b^	0.28±0.06^e^	0.71±0.23^d^
Liver	1.00±0.19^d^	1.23±0.17^bc^	2.03±0.12^a^	1.11±0.09^cd^	1.43±0.21^b^	0.74±0.08^e^	0.43±0.08^f^
14 days							
Duodenum	1.00±0.18^b^	0.83±0.15^bc^	1.72±0.28^a^	0.99±0.20^b^	0.58±0.23^c^	0.69±0.12^bc^	1.60±0.28^a^
Jejunum	1.00±0.23^a^	0.54±0.12^bc^	0.31±0.06^c^	1.20±0.36^a^	0.56±0.32^bc^	0.84±0.22^ab^	0.95±0.22^a^
Ileum	1.00±0.04^cd^	1.73±0.21^ab^	1.14±0.12^cd^	1.36±0.40^bc^	0.79±0.13^de^	0.44±0.11^e^	1.96±0.67^a^
Liver	1.00±0.12^bc^	0.49±0.08^cd^	0.66±0.04^cd^	1.31±0.20^b^	0.21±0.04^d^	0.22±0.08^d^	5.59±0.94^a^
21 days							
Duodenum	1.00±0.16^c^	1.94±0.26^a^	1.65±0.18^b^	0.85±0.10^cd^	0.62±0.04^d^	0.72±0.08^d^	0.99±0.14^c^
Jejunum	1.00±0.19^d^	2.42±0.24^b^	1.18±0.25^d^	3.87±0.30^a^	0.97±0.21^d^	0.85±0.25^d^	2.05±0.32^c^
Ileum	1.00±0.13^b^	1.43±0.17^a^	1.15±0.08^b^	0.47±0.09^d^	0.33±0.08^d^	0.75±0.20^c^	0.77±0.16^c^
Liver	1.00±0.10^d^	1.58±0.18^b^	1.87±0.34^a^	0.21±0.04^f^	0.35±0.11^f^	0.69±0.08^e^	1.27±0.16^c^

Means within columns with different superscript letters are different per p<0.05

### Development changes of FAS mRNA in yellow-feathered broilers

The relative expression levels of FAS mRNA in yellow-feathered broilers fed dietary bile salt supplements on 7, 14, and 21 days reveal several significant changes (Table-5). Compared with the negative control group, the expression of FAS mRNA in the duodenum decreased except for B5 on day 7. In addition, a decrease in B3 and B4 was observed on 14 days, and on 21 days, FAS mRNA decreased in B4 (p<0.05). When these were compared to the positive control on 7 days, the expression of FAS mRNA in the duodenum increased in B3 and B5, but on 14d, B2, B3, and B4 were markedly reduced (p<0.05). On day 21, B3 and B4 reduced the expression of FAS mRNA (p<0.05). When the expression of FAS mRNA was compared in the jejunum tissue with the experimental groups, we noted a significant decrease in B2 and B3 on 7 days (p<0.05). We observed decreases in expression in B1, B3, and B4 on 21 days (p<0.05). Comparisons among the positive control with the experimental groups adding bile salts showed that FAS mRNA expression in the jejunum decreased in B4 and B5 on day 14. On day 21, B1 and B4 decreased the expression of FAS mRNA (p<0.05). When the expression of FAS mRNA was compared between the positive control and experimental groups in the ileum, there was a decrease in B3 and B4 on 7 days; other BA groups except for B1 on day 14 and B1, B2 and B3 on day 21 (p<0.05). When these were compared to the positive control on 7 days, the expression of FAS mRNA in ileum increased in the bile salt supplement group, while reductions in B2, B3, and B4 were noted on day 14 (p<0.05). When compared with the negative control group, the expression of FAS mRNA in the liver was decreased in B5 on 7 days, and was also lowered in B1 and B4 on 14 days (p<0.05). Comparisons between the positive control group and the bile salt group showed decreased activity on 7 days except for B1 (p<0.05). On day 14**,** B1 and B4 showed reduced expression FAS mRNA (p<0.05).

**Table 5:** Relative expression of FAS mRNA on 7, 14, and 28-day-old yellow feather broiler chickens.

Treatments	A	C	B1	B2	B3	B4	B5
7 days							
Duodenum	1.00±0.18^a^	0.37±0.05^c^	0.59±0.14^bc^	0.59±0.07^bc^	0.61±0.14^b^	0.59±0.18^bc^	0.91±0.16^a^
Jejunum	1.00±0.23^c^	0.40±0.06^e^	0.77±0.07^cd^	0.55±0.06^de^	0.57±0.05^de^	2.64±0.31^a^	2.34±0.21^b^
Ileum	1.00±0.09^bc^	0.26±0.04^f^	0.79±0.10^cd^	1.10±0.23^b^	0.62±0.09^de^	0.49±0.07^ef^	1.91±0.37^a^
Liver	1.00±0.13^b^	2.36±0.39^a^	2.08±0.11^a^	0.97±0.30^b^	0.89±0.14^b^	0.72±0.21^b^	0.31±0.05^c^
14 days							
Duodenum	1.00±0.19^bc^	1.33±0.15^ab^	1.53±0.45^a^	0.73±0.23^cd^	0.52±0.04^d^	0.41±0.14^d^	1.37±0.24^a^
Jejunum	1.00±0.18^d^	1.35±0.18^c^	1.84±0.19^b^	1.84±0.13^b^	2.52±0.29^a^	0.88±0.13^d^	1.08±0.05^d^
Ileum	1.00±0.08^a^	0.97±0.10^ab^	0.85±0.08^ab^	0.34±0.11^d^	0.43±0.19^cd^	0.51±0.09^c^	0.82±0.07^b^
Liver	1.00±0.02^c^	1.06±0.18^c^	0.65±0.28^d^	1.83±0.33^b^	2.24±0.39^a^	0.68±0.11^d^	0.75±0.07^cd^
21 days							
Duodenum	1.01±0.13^c^	1.88±0.23^b^	1.45±0.21^bc^	1.79±0.40^b^	1.03±0.13^c^	0.48±0.04^d^	2.56±0.78^a^
Jejunum	1.00±0.22^ab^	0.74±0.06^c^	0.30±0.05^d^	0.81±0.17^bc^	0.73±0.20^c^	0.47±0.04^d^	1.20±0.22^a^
Ileum	1.00±0.14^b^	0.46±0.09^c^	0.53±0.31^c^	0.52±0.06^c^	0.39±0.05^c^	1.02±0.15^b^	1.56±0.29^a^
Liver	1.00±0.12^b^	0.46±0.28^b^	0.52±0.12^b^	0.51±0.10^b^	0.51±0.09^b^	0.50±0.05^b^	2.81±0.81^a^

Means within columns with different superscript letters are different per p<0.05

### Development changes of ACC mRNA in yellow-feathered broilers

Table-6 shows the changes in ACC mRNA relative expression levels in yellow-feathered broilers fed diets supplemented with bile salts on 7, 14, and 21 days of age. Compared with the negative control group, the expression of ACC mRNA in the duodenum decreased in the bile salt supplement group on 7 days and was also lowered in B1, B3, and B4 on 14 days (p<0.05). On 21 days, the expression of ACC mRNA was reduced in B3 and B4 (p<0.05). Compared to the positive control on 14 days, the expression of SREBF1 mRNA in the duodenum in B1, B3, and B4 was markedly reduced (p<0.05). On day 21, the bile salt supplement group decreased the expression of ACC mRNA (p<0.05). When the expression of ACC mRNA was compared between the negative control and experimental groups in the jejunum, a decrease was found in the bile salt supplement group on 7 days, B1, B3, and B5 on day 14 andB1 and B4 on day 21 (p<0.05). Comparisons between the positive control group and the bile salt group showed increases in activity on day 7 in all treatment groups except B5 (p<0.05). On day 14, B5 expression decreased, and on day 21, B1 and B4 reduced the expression of ACC mRNA (p<0.05). When the expression of ACC mRNA was compared between the negative control and experimental groups in the ileum, a decrease was found in B4 on 7 days, along with B1, B2, and B3 on day 14 and B2 and B3 on day 21 (p<0.05). Compared with the positive control group, B1 and B4 reduced the expression of ACC mRNA on 7 and 14 days and in the bile salt supplement group, we noted a reduction on 21 days; B2 also decreased the expression level of ACC mRNA (p<0.05). The levels of expression of ACC mRNA in the liver were reduced in B5 on day 7, and day 14, a reduction occurred in B4, while on day 21, a reduction was noted in the bile salt supplement group compared to the negative control (p<0.05). Comparisons between the positive control and bile salt supplemental group showed increases in activity on day 7 (p<0.05). On day 14, B4 showed decreased expression of ACC mRNA (p<0.05).

**Table 6:** Relative expression of ACC mRNA on 7, 14, and 28-day-old yellow feather broiler chickens.

Treatments	A	C	B1	B2	B3	B4	B5
7 days							
Duodenum	1.00±0.21^a^	0.18±0.03^c^	0.48±0.11^b^	0.56±0.03^b^	0.41±0.08^b^	0.25±0.04^c^	0.57±0.12^b^
Jejunum	1.01±0.12^a^	0.18±0.03^d^	0.81±0.11^b^	0.49±0.09^c^	0.48±0.02^c^	0.40±0.08^c^	0.22±0.04^d^
Ileum	1.00±0.06^bc^	1.13±0.10^b^	0.56±0.15^cd^	2.64±0.60^a^	1.24±0.48^b^	0.13±0.03^d^	0.83±0.12^bc^
Liver	1.00±0.08^bc^	0.58±0.13^e^	1.09±0.16^b^	1.60±0.11^a^	1.04±0.09^b^	0.87±0.09^cd^	0.75±0.05^d^
14 days							
Duodenum	1.00±0.10^a^	0.97±0.15^a^	0.59±0.15^bc^	0.91±0.15^a^	0.42±0.03^c^	0.66±0.15^b^	0.92±0.13^a^
Jejunum	1.01±0.04^a^	0.59±0.04^c^	0.44±0.21^cd^	0.82±0.08^a^	0.58±0.17^c^	0.96±0.17^a^	0.37±0.08^d^
Ileum	1.00±0.11^b^	1.63±0.21^a^	0.63±0.23^d^	0.69±0.07^cd^	0.23±0.03^e^	0.94±0.25^bc^	1.14±0.23^b^
Liver	1.00±0.05^b^	0.92±0.14^b^	1.26±0.49^ab^	1.51±0.03^a^	1.08±0.13^b^	0.42±0.11^c^	1.20±0.24^ab^
21 days							
Duodenum	1.00±0.06^bc^	3.77±0.53^a^	1.38±0.42^b^	0.82±0.01^cd^	0.60±0.06^d^	0.59±0.09^d^	1.07±0.16^bc^
Jejunum	1.00±0.02^ab^	0.71±0.13^b^	0.52±0.15^c^	0.80±0.15^bc^	1.28±0.24^a^	0.48±0.06^c^	1.06±0.48^ab^
Ileum	1.00±0.08^bc^	0.82±0.15^cd^	1.43±0.24^a^	0.42±0.09^e^	0.69±0.08^de^	1.29±0.35^ab^	1.18±0.19^ab^
Liver	1.00±0.05^a^	0.57±0.08^cd^	0.79±0.19^b^	0.72±0.12^bc^	0.74±0.14^b^	0.46±0.07^d^	0.51±0.08^d^

Means within columns with different superscript letters are different per p<0.05

## Discussion

BAs are the major components of bile, promoting the digestion, and absorption of fat in the small intestine and regulating cholesterol and energy homeostasis [18]. BAs are synthesized primarily in the liver and then secreted into the bile ducts, subsequently stored in the gallbladders of birds and many mammals. Ultimately, the bile is released into the small intestine, where approximately 95% of BAs are reabsorbed by passive diffusion and return to the liver through the portal vein. This is referred to as the BA enterohepatic circulation pathway. The enterohepatic circulation of BAs plays an important role in emulsifying and intestinal absorption of lipids and other nutrients [19,20]. It is now clear that BAs can also act as signaling molecules to regulate the metabolism of fat, glucose, and energy metabolism [21]. BAs regulate self-synthesis, and hepato-intestinal circulation and regulate triglycerides, cholesterol, glucose, and energy balance [22,23]. After hatching, a chick’s ability to digest and absorb fat is stunted because of limited bile secretion [24]. For this reason, synthetic BAs and bile salts have been studied in chick diets to improve fat digestion.

Studies have shown that BAs can activate Farsenoid X receptor, thus inhibiting the expression of SREBF1, FAS, and ACC in the liver [25,26]. Sterol-regulatory element transporter (SREBF) is a transcription factor belonging to the basic helical-loop-helical-leucine zipper family. It plays an important role in controlling cholesterol and fatty acid biosynthesis [27]. In addition, SREBF-1 can further catalyze the transcription of key genes required for fat syntheses, such as ACC and FAS [28]. FAS and ACC are key rate-limiting enzymes in the fatty acid synthesis pathway and play an indispensable role in the metabolic process of fat synthesis. Their activity and expression can regulate the synthesis of fatty acids, thus affecting fat metabolism and body fat deposition [12]. Among them, acetyl-CoA carboxylase ACC is the first step to catalyze the synthesis of fatty acids, and the synthesis of fatty acids into FAS is the last step in the pathway of catalyzing the formation of fats, and the key determinant of the maximum ability of the tissue to synthesize fatty acids [29]. Moreover, ACC and FAS have the highest correlation among adipogenesis genes related to adipogenesis [30].

This study shows that adding different levels of bile salt to the diet decreases the mRNA expressions of SREBF1, FAS, and ACC in the intestine and liver, which shows that bile salt supplementation might reduce fat synthesis to a certain extent. Previous studies have shown that dietary addition of 0.5% purified chenodeoxycholic acid reduces the expression levels of ACC-1c and FAS, inhibits feed intake, and reduces body weight in broilers [31]. In addition, Yin *et al*. [17] found that dietary bile salt supplementation could effectively inhibit the expressions of SREBP-1 and FAS in the liver. However, it upregulated the expressions of carnitine palmitoyltransferase 1 and peroxisome proliferator-activated receptors in the liver. Our results are similar to those of Ge *et al*. [16], who found that the addition of bile salt in the diet can down-regulate the expression levels of SREBF1, FAS, and ACC, such as adipogenic genes.

FATPs play an important role in the absorption and metabolism of long-chain fatty acids. FATP4 is the only member of the FATPs family that exists in the intestinal tract of animals. It is present in intestinal villus cells and plays an important role in regulating the absorption of fatty acids by intestinal cells [32,33]. Previous studies [34-36] have found that the expression of FATP4 mRNA is the highest in the small intestine, exists in the brain, liver, heart, skeletal muscle, and adipose tissue, and is expressed in these measured tissues. Wang *et al*. [37] found that the expression level of FATP4 mRNA in small intestine tissues of montane black bone chickens was higher than that in other tissues examined, and the expression level of FATP4 mRNA in various tissues showed significant developmental changes with the increase of age. In addition, the expression of FATP4 mRNA was the highest in the small intestine. In a somewhat similar outcome, we found that adding different bile salt levels could down-regulate the expression of FATP4 mRNA in the small intestine and liver.

The results obtained in this study suggest that different levels of bile salt supplementation in the diet of young chicks can regulate the expression of genes related to fat metabolism to promote fat catabolism and inhibit fat synthesis. The supplementation of bile salts plays a key role in improving fat deposition in broilers.

## Conclusion

Dietary supplements of bile salts play an important role in the development of young chicks. The observations documented in this study support this premise. Specifically, our results show that BA supplementation in the diet of yellow-feathered broilers had the best effect at 4.5 mg/kg. At this concentration, there is an effective regulatory expression of genes related to fat metabolisms, such as SREBF1, FAS, ACC, and FATP4 mRNA, to promote fat catabolism while inhibiting fat synthesis. In addition, the use of bile salt is a viable alternative to antibiotic use in the agricultural production of broilers. Understanding the metabolic dynamics of how dietary supplements can alter fat catabolism and synthesis is an important first step toward improving the environmental sustainability of broiler production, along with improving consumer safety. Therefore, it is necessary to study the effects of bile salts on the mechanisms of lipid metabolism to maximize the efficiency on which broilers chickens are grown.

## Authors’ Contributions

ZZ: He is a master’s student who did most of the research, contributed to data collection, analysis, and manuscript preparation. BD and SRM: Designed the research, method, data collection, and manuscript revision. HH: Data collection, analysis, and manuscript drafting. XL, JG, PL, and JW: Directed in method and data collection. All authors read and approved the final manuscript.
